# Subclinical Anthrax Exposure in Railroad Workers Following Soil Disruption in an Endemic Region: A Seroprevalence Study in Kars, Türkiye

**DOI:** 10.3390/pathogens15060644

**Published:** 2026-06-17

**Authors:** Ozgur Celebi, Hugh Dyson, Thomas R. Laws, Fatih Buyuk, Mehmet Doganay, Mitat Sahin, Les Baillie

**Affiliations:** 1 Department of Medical Microbiology, Faculty of Medicine, Kafkas University, Kars 36100, Türkiye; 2 Defence Science and Technology Laboratory, CBR Division, Porton Down, Salisbury SP4 0JQ, UK; 3 Department of Microbiology, Faculty of Veterinary Medicine, Kafkas University, Kars 36300, Türkiye; fatihbyk08@hotmail.com (F.B.); 4 Department of Infectious Diseases, Faculty of Medicine, Lokman Hekim University, Ankara 06530, Türkiye; 5 Faculty of Veterinary Medicine, Kyrgyz-Turkish Manas University, Chingiz Aitmatov Campus, Djal, Bishkek 720038, Kyrgyzstan; 6 Department of Microbiology, School of Pharmacy and Pharmaceutical Sciences, Cardiff University, Cardiff CF10 3NB, UK

**Keywords:** *Bacillus anthracis*, seroprevalence, Kars region, occupational exposure, railroad workers, subclinical infection

## Abstract

During construction of the Baku–Tbilisi–Kars railroad between Kars City in Türkiye and Tbilisi in Georgia, blasting operations in an anthrax-endemic region disrupted a burial pit containing carcasses of cattle that had died of anthrax. Railroad workers expressed concerns that release of this material could result in them developing anthrax infection. We therefore undertook a seroprevalence study six months later to seek evidence of exposure to *Bacillus anthracis* spores. We used an optimised Enzyme-Linked Immunosorbent Assay (ELISA) to screen serum for antigen-specific IgG antibodies to the anthrax toxin subunits Protective Antigen (PA) and Lethal Factor (LF). Stepwise linear regressions and *t*-tests were performed to compare results from railroad workers (n = 64) with a group of long-term Kars City residents (urban dwellers, n = 16), who had no history of possible contact with anthrax antigens. Anti-PA IgG concentrations were higher (*p* = 0.038) in railroad workers than in urban dwellers, but anti-LF IgG concentrations did not differ (*p* = 0.932) between the two groups. The anti-PA response is known to be dominant, and the difference was small. The lack of LF response did not preclude an antibody response to *B. anthracis*. Following the blasting operations, no cases of anthrax infection occurred in either railroad workers or villagers living nearby, suggesting that the spore exposure (evidenced by higher antibody titres) was at levels insufficient to initiate clinical infection. The elevated PA-specific antibody responses in railroad workers compared with urban dwellers might be consistent with the former having had previous subclinical exposure to *B. anthracis*. In anthrax-endemic regions, therefore, construction activities that involve blasting or large-scale excavation may pose risks of occupational exposure to *Bacillus anthracis* spores.

## 1. Introduction

Humans primarily contract anthrax, caused by *Bacillus anthracis*, from exposure to spores from infected animals or contaminated animal products. However, it has been shown in several occupational settings that working in an environment where contact with spore-contaminated material is likely does not necessarily lead to clinical infection. This principle is illustrated by results of studies in a classic industrial setting for occupational anthrax (traditionally known as Wool-sorter’s Disease): textile mills that process untreated animal hair.

Surveys in a New Hampshire textile mill in 1957 following an outbreak of clinical anthrax infection [1,2,3], and in a Belgian wool-cleaning factory in 2006–2007 for a planned occupational risk assessment [1,4,5], demonstrated several common features. Both factories processed untreated goat hair and (sheep) wool from parts of the world where anthrax is endemic, and viable anthrax spores were isolated from air [2], air-filter dust [4], goat-hair [2,4] and wool [4]. During a 10-week period in 1957, five cases of inhalational and four of cutaneous anthrax occurred in the New Hampshire mill. In both factories, positive serum antibody responses to Protective Antigen, a subunit of the *B. anthracis* toxin complex [1,6,7], were found in workers with no history or clinical signs of infection. In the New Hampshire mill, there was a possible relationship between ambient spore levels and seropositivity [2], in that seven of the 11 individuals who were seropositive three months after the outbreak worked in the combing–carding area of the plant where six of the nine clinical cases had worked. Similarly in the Belgian mill, 30% of workers processing and 20% of those sorting raw goat hair were seropositive, compared to only 3% in less exposed roles [1].

We recently reported a study [8] measuring anti-PA and anti-LF IgG antibodies in serum from 279 healthy individuals living in the anthrax-endemic Kars region of eastern Türkiye, 105 of whom had previously had clinically confirmed anthrax infections. Linear discriminant analysis of antibody concentrations from these 105 individuals was used to identify profiles associated with known prior infection. The remaining 174 participants had no history of infection, but 72 of them had a documented history of exposure to anthrax-contaminated materials. Group analysis of antibody profiles from these 174 individuals showed that profiles typical of previous infection were present and were positively associated (*p* = 0.002) with duration of continuous exposure risk, implying that longer exposure to anthrax spores is linked to a higher probability of unrecognised infection. In addition, four specific individuals amongst this group had antibody concentrations strongly suggestive of previous *B. anthracis* infection; two were butchers, and two were rural dwellers whose main occupation was animal husbandry. The most likely sources of infectious spores would have been contaminated meat for the butchers, and sick animals or possibly contaminated soil in the local environment for the rural dwellers.

Although anthrax spores can persist in soil for prolonged periods, clinically confirmed infections attributable to direct soil contact are rarely reported [9]. The actual frequency of human infection due to contaminated soil is unknown; however, the risk is discussed in considerable detail [10]. Nevertheless, and while entirely anecdotal, a national UK newspaper article [11] reported that Russian troops contracted anthrax while digging trenches in the Zaporizhzhia region of Ukraine, thus illustrating the public interest in this bacterium. This suggestion is at odds with a review [11] that concluded there is no scientific evidence that anthrax infections have occurred in military animals or soldiers from exposure during major soil disruption in known endemic areas.

**Figure 1:**
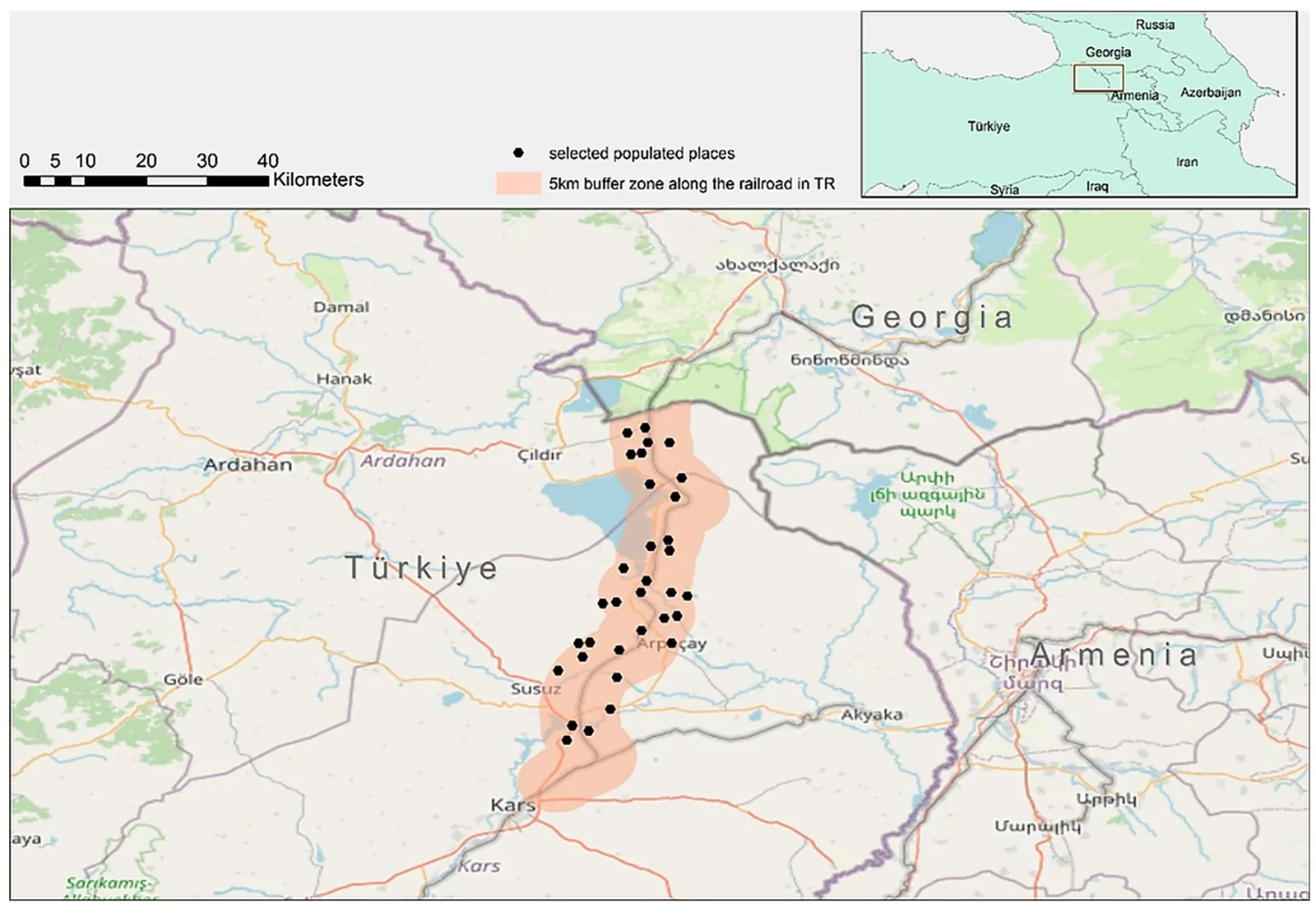
Map showing the Kars City to Tbilisi section of the BTK railroad. Province and district names are presented in their official Turkish spelling. © Kafkas University copyright (2026).

An opportunity arose during construction of the Kars City to Tbilisi section of the Baku–Tbilisi–Kars (BTK) railroad [12] to explore this question further and assess whether health impacts could arise from excavating spore-contaminated soil. The railroad route runs through anthrax-endemic terrain north-east of Kars City, near villages where bovine anthrax had previously occurred and infected animal carcases were buried without appropriate decontamination (Figure 1 shows the railroad route relative to endemic villages and the identified burial site) [13]. Construction operations at several locations during October 2013 involved blasting rocks and the excavation of large quantities of earth. One burial site, which consisted of shallow graves containing carcasses of two cows that had died of anthrax early in 2012, was completely disrupted with soil and its contents spread over a wide area (MS, FB, and personal observations).

Concerns were expressed by railroad workers involved in the construction who feared they might develop anthrax infection, and local villagers who worried that any contaminated material released could cause severe illness in their livestock. No cases of anthrax were, however, identified around this time amongst railroad workers, local people, or their livestock. In order to explore the possibility that subclinical exposures might have occurred, serum samples were collected from a group of railroad workers in April 2014 to screen for antibodies specific for *B. anthracis* toxins. This paper describes the results of this study and sheds light on occupational risks of spore exposure during major construction projects in anthrax-endemic regions.

## 2. Materials and Methods

### 2.1. Patient and Public Involvement

This study was carried out in response to concerns expressed both by railroad workers involved in the construction of the BTK railroad through anthrax-endemic terrain and by residents of villages along the railroad route. Both groups were concerned about the potential health implications of the release of contaminated material from animal burial sites. The railroad workers were concerned that they themselves might fall ill, while the villagers were particularly worried that their livestock might be affected. Representatives of both groups, acting as informal ad hoc community representatives, contacted the Kafkas University Veterinary Microbiology Department about their concerns and discussed the likely risks of community exposure with university veterinarians. The concept of this study grew out of these discussions, and the serological survey was designed, arranged and carried out in collaboration between these representatives and university veterinarians, in order to address community concerns in a transparent, easily understood and structured manner.

### 2.2. Study Background and Design

The primary objective was to ascertain whether railroad workers were exposed to *B. anthracis* spores during construction of the Kars City to Tbilisi section of the BTK railroad [11]. Whilst the area is endemic for *B. anthracis* spores, only one site with contamination previously confirmed by microbiological culture was known to have been disrupted by the construction activities. This was a burial pit containing the carcasses of two cows that had died of anthrax early in 2012; the pit was located near a cart track nearly 2 km from a village in Arpaçay District [13]. The carcasses had been buried in shallow graves one metre apart without prior decontamination, and covered with superficial mounds of earth (FB, personal communication).

Soil samples were taken by Kafkas University veterinary microbiologists from three positions at this site on four occasions between July 2012 and August 2013 (Table 1), for the Department’s anthrax surveillance programme. The standard envelope sampling method used for large sites was adapted due to the irregular nature of this site. Sample Positions 1 and 2 were situated above the first carcass, with Position 1 at the centre of the mound and Position 2 located 0.5 m to the east. Position 3 was at the centre of the mound above the second carcass, 1 m from Position 1. Soil sampling procedures and methods for *B. anthracis* culture and identification have previously been described in detail [13].

For the serological study, volunteers aged 18 years or older were sought from the group of construction workers involved in the Kars City to Tbilisi section of the BTK railroad, and from a comparator group of healthy individuals living in Kars City who had never worked on railroads. Following a briefing on the study’s purpose and nature, fully informed consent was given by each of 64 railroad workers and 16 Kars City residents (classified as urban dwellers). Blasting operations in the study area took place during October 2013 and early November 2013. Blood samples were subsequently collected from railroad workers on 24–25 April 2014 and from urban dwellers on 29 July and 14 August 2014, corresponding to approximately 5–6 months and 8–10 months after the blasting activities, respectively. Whole-blood samples (15 mL) were collected from all individuals by venipuncture. After centrifugation, serum was separated, divided into aliquots, and maintained at −80 °C until laboratory testing.

### 2.3. Demographic and Clinical Information

The 64 railroad workers were males whose mean age was 37.5 years (range 23–61). The urban dweller group comprised 11 males and five females with a mean age of 24.4 years (range 22–26). Inclusion of females in the comparator group was considered acceptable since no gender differences in anti-anthrax toxin antibody concentrations had been found in the serology study involving 279 individuals that we undertook in the Kars region during 2012–2013 [8]. No study participant had been vaccinated against anthrax or had any history of anthrax or anthrax-like infection; furthermore, none of them had been in other identified situations where exposure to anthrax spores might have occurred. In addition, individuals employed in occupations associated with an increased risk of anthrax exposure, including veterinarians, livestock breeders, butchers, agricultural workers, or other occupations involving regular contact with animals or animal products, were not included in the comparator group.

The railroad workers had been employed on railroad construction in Türkiye for a mean of 11.8 years (range 1.0–36) and on the Kars City to Tbilisi section of the BTK railroad for a mean of 3.5 years (range 0.2–6.0), where they had undertaken one of five different roles. There were 30 drivers of large earth-moving trucks, 28 operators of mechanical diggers, four foremen responsible for workforce supervision, one member of office staff, and one person whose job was to drill holes to place explosives for blasting. Railroad workers were based either at the main supply depot (n = 8) or one of three segments (n = 11, 19, 26) of the Kars City to Tbilisi section of the BTK railroad.

### 2.4. Serological Analyses

#### 2.4.1. Enzyme-Linked Immunosorbent Assay (ELISA)

Antigen-specific IgG antibodies against PA and LF were quantified by indirect ELISA, as previously described [8]. Ninety-six-well microplates (Code: BSD012, Biosigma S.r.l, Coma, Italy) were coated in columns 1–10 with 100 µL of recombinant antigen diluted to 2 µg/mL in phosphate-buffered saline (PBS). Recombinant Protective Antigen [PA] (Lot 17115A5B) and Lethal Factor [LF] (Lot 1722B11B) were purchased from Quadratech Diagnostics Ltd. (Epsom, Surrey, UK). Human IgG standards (Code: I4506, Sigma Aldrich, Dorset, UK) were included in columns 11 and 12 at concentrations of 0.5, 0.25, 0.125, and 0.0625 µg/mL, with each concentration analysed in triplicate. After antigen coating, plates were washed three times with PBS + 0.1% Tween-20 (PBST) using an automated microplate washer (Code: N15777, Thermo Scientific, Vantaa, Finland) and subsequently blocked for 1 h at 37 °C. Serum samples, positive control serum, and negative control serum were thawed prior to analysis. Samples were distributed across columns 1–8, positive controls in column 9, and negative controls in column 10. Serial two-fold dilutions were prepared in PBST beginning with a 1:10 dilution in the first well, following a previously described protocol [8] designed to enhance the detection of low antibody concentrations. Negative-control serum was obtained from healthy individuals residing in a non-endemic region with no known history of anthrax exposure. Following sample incubation, plates were washed three additional times with PBST. Bound antibodies were detected using a mouse monoclonal anti-human IgG antibody (Code: 209-035-088, Jackson ImmunoResearch Lab. Inc., Cambridgeshire, UK), diluted 1:1000 in PBST. After adding 100 μL conjugate solution per well, plates were incubated for 1 h at 37 °C and subsequently washed three times. For colour development, an ABTS substrate solution was prepared by dissolving 0.7 g sodium phosphate dibasic (Code: S5136-100G, Sigma, St. Louis, MO, USA) and 0.5 g citric acid (Code: 27102, Sigma, MO, USA) in 100 mL of deionised water. One ABTS (2,2′-azino-bis(3-ethylbenzothiazoline-6-sulphonic acid)) tablet (Code: A9941, Sigma, MO, USA) was added to the buffer, and hydrogen peroxide was incorporated immediately before use (2.5 µL H_2_O_2_ (Code: H1009-100ML, Sigma, MO, USA) per 10 mL substrate solution). A volume of 100 µL substrate was dispensed into each well, and plates were incubated at 37 °C for 30 min. The enzymatic reaction was terminated by adding 100 µL 2% SDS (Code: L4390-100g, Sigma, MO, USA). Optical density values were measured at 405 nm using a SpectraMax Plus384 microplate reader (Molecular Devices, Silicon Valley, CA, USA).

#### 2.4.2. Determination of IgG Concentration

Each serum sample was analysed in duplicate or triplicate for both antigens. Replicate measurements were performed in separate assay runs to minimise systematic bias and ensure that experimental variation was distributed randomly across analyses. An Excel macro was written to batch process results. For each sample, the program identified the first dilution yielding an optical density (OD) value below 0.6, corresponding to the central region of the assay’s linear detection range. The OD value obtained from the negative-control serum at the corresponding dilution was then subtracted to correct for background reactivity. Antibody concentrations were estimated by interpolation from a standard curve generated through linear regression of the mean OD values obtained from triplicate human IgG standards. The calculated concentrations from replicate measurements were subsequently averaged to obtain a single value for each serum sample and antigen combination. Final antibody concentrations were reported as μg/mL.

### 2.5. Statistical Analyses

Data management and preliminary processing were performed in Microsoft Excel prior to statistical evaluation. Statistical analyses were conducted using IBM SPSS Statistics version 27.1 (New York, NY, USA), which was used to generate descriptive demographic summaries and to perform stepwise linear regression and independent-samples *t*-test analyses. Graphical representations of the results were produced using GraphPad PRISM version 9.0. Categorical predictor variables were converted into binary dummy variables before inclusion in the statistical models. The distributional characteristics of continuous variables were assessed using quantile–quantile (Q–Q) plots, which indicated that parametric analytical approaches were appropriate. Measurements obtained from both ELISAs exhibited a positively skewed distribution and were therefore log10-transformed prior to analysis. Due to a control-based adjustment, two mean anti-LF IgG ELISA results were negative, so the transformation included the addition of a correction factor slightly greater than the minimum value for each set of results, according to the formula x = log_10_(x + 0.7).

## 3. Results

### 3.1. B. anthracis Spore Concentrations in Soil Samples

Soil samples were taken on four occasions between July 2012 and August 2013 from the contaminated animal burial site prior to its disruption in October 2013. The presence of *B. anthracis* spores in these samples was confirmed by microbiological examination; counts varied from 133 to 29,300 spores per gram of soil (Table 1) [13].

In 2014, following completion of the Kars City to Tbilisi section of the BTK railroad, a case of bovine anthrax was reported in another village along the railroad route; two environmental contamination sites in this village also tested positive for *B. anthracis* at this time. More recently, in 2021 and 2023, two cases of bovine anthrax in two other villages on the railroad route were confirmed by laboratory analysis. No cases of human anthrax were reported amongst local people throughout this time; however, these observations were based on routine passive surveillance and official disease notifications.

### 3.2. Specific IgG Antibody Responses to B. anthracis Toxins

**Figure 2:**
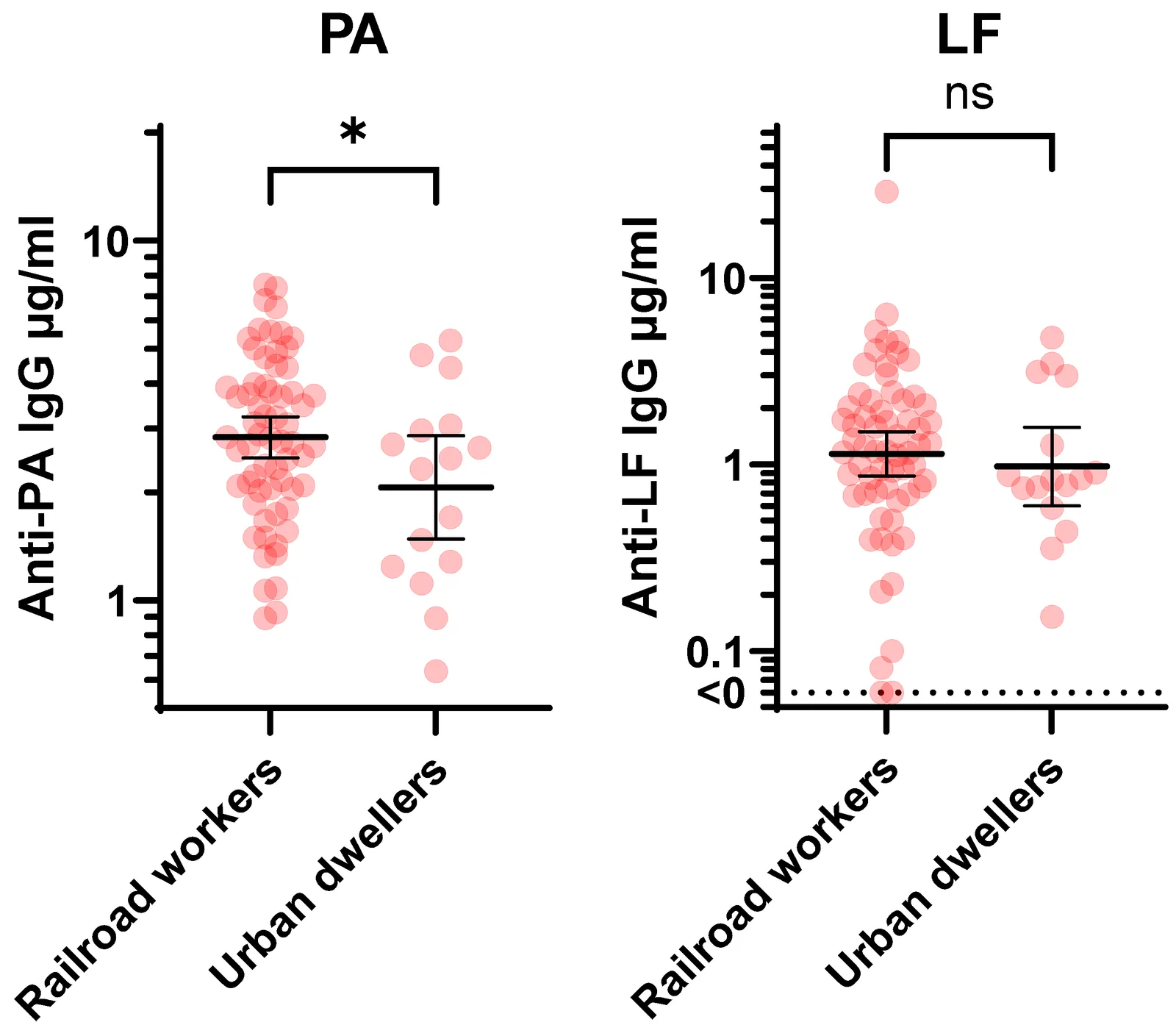
Anti-PA and Anti-LF IgG concentrations in railroad workers and urban dwellers. Each data point represents the concentration of a single participant. The concentration was determined through 2–3 replicate ELISAs. The line represents the geometric mean, and the error bars indicate the geometric 95% confidence interval. Significance of the difference between means was assessed by a *t*-test and is indicated by * = *p* < 0.05. The mean anti-PA IgG concentration was greater (*p* = 0.038) for railroad workers than for urban dwellers, but there was no evidence of any difference in anti-LF IgG concentrations between the two groups (*p* = 0.932).

The PA- and LF-specific antibody responses of the 64 railroad workers and 16 urban dwellers are compared in Figure 2 with summary statistics listed in Table 2.

Log_10_-transformed antibody concentrations in serum from railroad workers and urban dwellers were compared by t -test (Table 3), after a Levene’s test for unequal variance and examination of residuals had confirmed that a standard t -test was fit for purpose (Table 4).

The mean anti-PA IgG concentration was greater (*p* = 0.038) for railroad workers than for urban dwellers (Figure 2; Table 4), but there was no evidence of any difference in anti-LF IgG concentrations between the two groups (*p* = 0.932).

### 3.3. Analyses of Railroad Worker Occupational Subgroups

A statistical modelling approach was taken for the purpose of understanding whether differences in epidemiological data collected might have influenced the group-level difference in ELISA measurements. Antibody concentration datasets for the 64 railroad workers were analysed by separate stepwise linear regressions with Log_10_ anti-PA and Log_10_ anti-LF IgG concentrations as dependent variables. Stepwise model building was performed using F-tests to assess improvements between competing models. Entry and removal criteria were set at *p* < 0.05 and *p* > 0.15, respectively, and five explanatory variables were considered during model development. These were: (1) Railroad construction role [Truck driver (n = 30), digger operator (n = 28), foreman (n = 4), office staff (n = 1)], and blast hole driller (n = 1); (2) age; (3) time worked on railroad construction in Türkiye overall; (4) time worked on construction of Kars City to Tbilisi section of BTK railroad; and (5) part of BTK railroad worked on (one of three different railroad segments or main supply depot). None of these predictors was significant for either IgG antibody concentration at the *p* < 0.05 level.

## 4. Discussion

Construction of the BTK railroad through the anthrax-endemic Kars region involved extensive excavation and blasting operations, which disrupted at least one known contaminated animal burial site, and probably brought some railroad workers into close contact with *B. anthracis* spores. The fact that none reported any symptoms or signs of infection suggests that any exposures that did occur were at levels insufficient to initiate clinical infection. We did, however, observe significant elevations in anti-PA IgG antibody responses in railroad workers compared to local urban dwellers, which might be consistent with some in the former group having been previously exposed to this anthrax toxin antigen.

Local control groups in studies such as these are important, as anti-PA antibody concentrations in individuals with no history of previous infection or exposure to spore-contaminated materials vary according to the degree of anthrax endemicity in their local environment. In a study in India, healthy control individuals from anthrax-endemic regions had higher anti-PA and anti-LF IgG levels as assayed by ELISA than healthy controls from non-endemic regions [14]. However, Western blot analysis and competitive inhibition assays suggested that these elevated values were not due to increased anti-PA IgG levels, suggesting possible cross-reactivity with other antigens in the ELISA used. In a study in central Türkiye [15], serum samples from 20 healthy individuals with no history of anthrax infection, living in the anthrax-endemic Kayseri region, contained antibodies to PA and other components of the *B. anthracis* toxin complex. Some of these individuals had no toxin-neutralising activity but had IgG binding to toxin components similar in degree to those of 46 patients with documented cutaneous anthrax [16]. These authors suggested that some of this binding activity could be due to prior subclinical *B. anthracis* exposures or to unknown cross-reactive environmental exposures common locally. Interestingly, the antibody responses of these local Kayseri controls were significantly greater than those of 120 healthy US-based volunteers living in an anthrax-free region [15].

The ELISA method used in this study (and previously in [8]) has no defined positivity/negativity cut-off. As a continuous variable, the higher concentrations of antigen-specific antibody provide increased confidence of antigen-specific responses. In this study, antigen-specific responses are generally low (<10 µg/mL) with no obvious bimodality. These observations could suggest that the results might be negative and that the antigen–antibody binding observed was not a result of exposure to *B. anthracis*. Alternatively, they may indicate that the serological response in the test group was low and only slightly above background.

One possible explanation for these regional differences in anti-PA antibody concentrations in healthy volunteers is cross-reactivity between PA and pore-forming components of toxins produced by other pathogenic or environmental bacteria [15]. PA has significant amino acid sequence homology with the pore-forming components of *Clostridium perfringens* iota toxin and toxins produced by *Clostridium spiroforme*. Cross-reactivity between these Clostridial proteins and PA has been demonstrated in Western blot assays but not in ELISAs, suggesting that the Clostridial pore-forming proteins are immunologically distinct from PA produced by *B. anthracis* [16]. Some of these apparent discrepancies could also be due to different characteristics of the ELISAs or Western blots used by different laboratories, which emphasises the benefit of internationally recognised standard assays for key bacterial antigens, which are unfortunately not yet available for PA. Another possible explanation for the regional differences observed in these two studies [13,14] is that anthrax toxin antigens in the environment of anthrax-endemic regions could generate an immune response from unrecognised low-dose exposures. Indeed, we found that individuals living in rural parts of the Kars region can have detectable circulating anti-anthrax toxin antibody concentrations, despite having no history of anthrax infection, vaccination or contact with potentially contaminated material [8].

Whatever the cause of these regional differences, however, the groups of railroad workers and urban dwellers in our study are both likely to have had similar prior exposures to environmental anthrax spores and to any other potentially cross-reacting antigens that might exist in their local environment. We therefore concluded that the railroad workers had previously been exposed to anthrax toxin antigens. The only candidate mechanism of such an exposure that we have been able to identify is the disruption of soil containing *B. anthracis* spores during railroad construction through the anthrax-endemic part of Kars province, but it has not been possible to determine the timing of any exposure from the data available.

Information regarding the timing of an exposure to anthrax toxin antigens can be gained by considering the time courses of antibody responses to PA and LF, as these differ both during cutaneous infection [17] and also following booster vaccination [18]. In both these contexts, responses to LF appeared earlier and declined sooner over subsequent months than did the responses to PA; this trend was particularly apparent during the 4 months following booster doses of the UK anthrax vaccine [18]. Our findings of baseline anti-LF but elevated anti-PA antibody concentrations in the railroad workers are therefore not inconsistent with prior exposure to low doses of spores. However, without sequential serum samples before and during an individual’s employment in railroad construction, a point in time at which seroconversion occurred cannot be identified with certainty. That being said, there were no reported cases of anthrax infection amongst the construction workers during the 10 years in which the BTK railroad was being built, and no individuals participating in this study had previously had any other identified contact with anthrax-contaminated material. That leaves the possibility that some were exposed to low levels of anthrax spores whilst moving earth or blasting through terrain contaminated with viable *B. anthracis* spores [13].

Clarification of these questions will require further epidemiological studies in anthrax-endemic parts of the world, but our view is that transport infrastructure projects in these regions should be regarded as occupations that entail a risk of *B. anthracis* spore exposure. Probably the most important negative aspect of this study, however, is that the anthrax infections in humans and livestock, feared respectively by railroad workers and local villagers, did not materialise. We concluded that the spore exposure (evidenced by higher antibody concentrations) was at levels that were insufficient to initiate clinical infection.

## 5. Conclusions

(1) The elevated PA-specific antibody responses observed in the railroad workers compared with the urban dwellers might be consistent with some railroad workers having had prior subclinical exposure to *B. anthracis* spores.

(2) The fact that no railroad workers had ever had clinical signs of infection suggests that humans can work without clinical illness in environments contaminated with *B. anthracis* spores.

(3) In anthrax-endemic regions, major construction projects involving blasting or other terrain disruption may disseminate viable spores and potentially cause occupational *B. anthracis* exposure.

(4) Burial alone does not eliminate spores, which may persist in soil for many years [11]. It is therefore important that infected animal carcasses are appropriately decontaminated before burial, in order to reduce the likelihood of viable spore dissemination during major construction projects. The potential for reactivation of dormant spores, due to changes in climate in anthrax-endemic regions, underlines the importance of effective decontamination [17].

## 6. Strengths and Limitations

The Veterinary Microbiology Department of Kafkas University has had an established anthrax surveillance programme in the Kars region for over 30 years, developing long-standing community engagement and trust. This gave local village leaders and railroad workers’ representatives the confidence to approach university veterinarians with their concerns regarding contaminated material released from anthrax burial sites by construction activities that could potentially cause sickness in humans and livestock. This long-established link between the university and village communities in the Kars region also enabled significant co-operation between researchers and participants in developing the concept of this study, as well as in its design and conduct.

This was a cross-sectional study, reporting anti-anthrax toxin antibody concentrations in blood samples given by a group of railroad workers and a comparator group of local urban dwellers, thereby minimising the risk of false-positive ELISA results in the former group, due to cross-reaction with locally present environmental antigens. None of the participants in the study had any history of recognised exposure to anthrax-contaminated material or illness consistent with anthrax infection. However, antibody concentrations in railroad workers were compatible with a previous low-level exposure, although in the absence of sequential samples over several years, it was not possible to identify when such an exposure might have occurred. Furthermore, retrospective information regarding personal protective equipment (PPE) use, duration of exposure, frequency of blasting activities, and exposure intensity during construction work was unavailable. Consequently, the impact of these factors on individual anthrax exposure risk and antibody responses could not be assessed. While the participants did not report any previous risk of exposure to *B. anthracis*, this cannot be wholly discounted as a possibility.

It needs to be acknowledged that there may be limitations in the comparator group. While stepwise statistical modelling did not imply that differences in age and sex between groups were likely to influence outcome, the sample size was small and therefore cannot be discounted. For example, only one “blast hole driller” was enlisted, making any investigations of this role statistically impossible. Moreover, ideally, a nearly equal number of people within each group would have elicited greater statistical power.

Our conclusions were limited by the absence of recognised international standard positive controls for the ELISAs, which would have enabled comparisons with assays conducted at different times in other laboratories. International standards would also have made possible comparison of the assay results in this study with those obtained previously in our laboratory. Thus, the railroad workers’ data could have been added as another occupational group to the large seroprevalence study in the Kars region that we have reported previously. A further limitation of the ELISA method was that the baseline removed generated two negative results. This might be indicative of lower-than-expected assay precision. In addition, although the in-house ELISA was based on a previously published protocol, detailed validation parameters, including assay specificity, reproducibility, and inter-assay variability, were not formally evaluated in the present study. Our conclusions are further limited by the lack of confirmatory Western blot or toxin neutralisation assay (TNA). However, it is noteworthy that the Clostridial cross-reactivity study [16] showed no ELISA cross-reactivity, thus strengthening the argument that the ELISA results presented here are true positives.

## Figures and Tables

**Table 1:** Anthrax spore concentrations in soil samples collected from burial site before blasting operations.

Sampling Date	Spore Concentration (CFU/g Soil)		
	Sample Position 1	Sample Position 2	Sample Position 3
July 2012	1363	Not done	Not done
May 2013	199	2330	266
June 2013	29,300	266	Not done
Aug 2013	133	266	None

None = no detectable *B. anthracis* colonies (0 CFU/g).

**Table 2:** Anti-PA and Anti-LF IgG concentrations in railroad workers and urban dwellers (raw untransformed data).

	Group	N	Minimum	25% Centile	Median	75% Centile	Maximum	Range
Anti-PA IgG (µg/mL)	Railroad workers	64	0.8907	2.043	2.828	3.974	7.528	6.637
	Urban dwellers	16	0.6343	1.250	2.398	3.033	5.271	4.637
Anti-LF IgG (µg/mL)	Railroad workers	64	−0.6961	0.7034	1.214	2.168	28.96	29.65
	Urban dwellers	16	0.1529	0.6286	0.8355	2.558	4.798	4.645

**Table 3:** Anti-PA and Anti-LF IgG concentrations (Log_10_ + 0.7 transformed data).

	Group	N	Mean	S. D.	S. E. M.	*t*-Test (2-Tailed)
Log Anti-PA IgG	Railroad workers	64	0.4529	0.2264	0.0283	*p* = 0.038
	Urban dwellers	16	0.3138	0.2691	0.0673	
Log Anti-LF IgG	Railroad workers	64	0.2722	0.4521	0.0565	*p* = 0.932
	Urban dwellers	16	0.2622	0.2357	0.0589	

**Table 4:** Independent samples tests for IgG antibody concentrations in railroad workers and urban dwellers.

	Levene’s Test—For Equality of Variances		*t*-Test—For Equality of Means						
		Sig.			Sig. (2-Tailed)	Mean Difference	Std. Error Difference	95% CI of Difference	
	F	*p*	T	Df	*p*			Lower	Upper
Log Anti-PA IgG	1.250	0.267	2.115	78	0.038	0.1390	0.0657	0.0082	0.2699
Log Anti-LF IgG	0.366	0.547	0.085	78	0.932	0.0100	0.1172	−0.2233	0.2433

## Data Availability

Data used for this report are contained within the article.

## Abbreviations

The following abbreviations are used in this manuscript:

PA	Protective antigen
LF	Lethal factor
PBST	Phosphate-buffered saline + Tween 20
ABTS	2,2′-azino-bis(3-ethylbenzothiazoline-6-sulphonic acid
BTK railroad	The Baku–Tbilisi–Kars Railroad
