# Correction: Celebi et al. Subclinical Anthrax Exposure in Railroad Workers Following Soil Disruption in an Endemic Region: A Seroprevalence Study in Kars, Türkiye. *Pathogens* 2026, *15*, 644

**DOI:** 10.3390/pathogens15080808

**Published:** 2026-07-31

**Authors:** Ozgur Celebi, Hugh Dyson, Thomas R. Laws, Fatih Buyuk, Mehmet Doganay, Mitat Sahin, Les Baillie

**Affiliations:** 1 Department of Medical Microbiology, Faculty of Medicine, Kafkas University, Kars 36100, Türkiye; ozgurcelebi36@hotmail.com; 2 Defence Science and Technology Laboratory, CBR Division, Porton Down, Salisbury SP4 0JQ, UK; ehdyson@dstl.gov.uk (H.D.); trlaws@dstl.gov.uk (T.R.L.); 3 Department of Microbiology, Faculty of Veterinary Medicine, Kafkas University, Kars 36300, Türkiye; fatihbyk08@hotmail.com (F.B.); mitats@hotmail.com (M.S.); 4 Department of Infectious Diseases, Faculty of Medicine, Lokman Hekim University, Ankara 06530, Türkiye; mehmet.doganay@lokmanhekim.edu.tr; 5 Faculty of Veterinary Medicine, Kyrgyz-Turkish Manas University, Chingiz Aitmatov Campus, Djal, Bishkek 720038, Kyrgyzstan; 6 Department of Microbiology, School of Pharmacy and Pharmaceutical Sciences, Cardiff University, Cardiff CF10 3NB, UK

## Change in Corresponding Author

In the original publication [1], there was an error regarding the corresponding author information. The corresponding author has been changed from Fatih Buyuk to Les Baillie. The authors state that the scientific conclusions are unaffected. This correction was approved by the Academic Editor. The original publication has also been updated.
